# What variables are important in predicting bovine viral diarrhea virus? A random forest approach

**DOI:** 10.1186/s13567-015-0219-7

**Published:** 2015-07-24

**Authors:** Gustavo Machado, Mariana Recamonde Mendoza, Luis Gustavo Corbellini

**Affiliations:** Laboratory of Veterinary Epidemiology, Faculty of Veterinary, Federal University of Rio Grande do Sul (UFRGS), Av. Bento Gonçalves 9090, CEP 91540-000 Porto Alegre, RS Brazil; Experimental and Molecular Cardiovascular Laboratory, Experimental Research Center, Hospital de Clínicas de Porto Alegre (HCPA), Av. Ramiro Barcelos, 2350, CEP 99010-115 Porto Alegre, RS Brazil

## Abstract

Bovine viral diarrhea virus (BVDV) causes one of the most economically important diseases in cattle, and the virus is found worldwide. A better understanding of the disease associated factors is a crucial step towards the definition of strategies for control and eradication. In this study we trained a random forest (RF) prediction model and performed variable importance analysis to identify factors associated with BVDV occurrence. In addition, we assessed the influence of features selection on RF performance and evaluated its predictive power relative to other popular classifiers and to logistic regression. We found that RF classification model resulted in an average error rate of 32.03% for the negative class (negative for BVDV) and 36.78% for the positive class (positive for BVDV).The RF model presented area under the ROC curve equal to 0.702. Variable importance analysis revealed that important predictors of BVDV occurrence were: a) who inseminates the animals, b) number of neighboring farms that have cattle and c) rectal palpation performed routinely. Our results suggest that the use of machine learning algorithms, especially RF, is a promising methodology for the analysis of cross-sectional studies, presenting a satisfactory predictive power and the ability to identify predictors that represent potential risk factors for BVDV investigation. We examined classical predictors and found some new and hard to control practices that may lead to the spread of this disease within and among farms, mainly regarding poor or neglected reproduction management, which should be considered for disease control and eradication.

**Electronic supplementary material:**

The online version of this article (doi:10.1186/s13567-015-0219-7) contains supplementary material, which is available to authorized users.

## Introduction

Bovine viral diarrhea virus (BVDV) has a single-stranded, positive-sense RNA genome and belongs to the genus *Pestivirus* of the family *Flaviviridae* [1], causing one of the most common and economically important viral diseases of cattle [2]. Several BVDV control strategies have been proposed and launched in many countries based on information about prevalence, incidence and associated risk factors, which is the baseline knowledge required for designing and implementing effective regional control actions [3].

A number of studies based on traditional risk factors identification approaches (logistic regression mainly) have been performed on BVDV [4-8], and the knowledge about major risk factors are related to the following: biosecurity [6], reproduction management [2,6,9,10], herd size [5,8], animal introduction [2,4,5,11], direct contact with other animals (from the same species or not) [4,11-13], communal grazing [4,5], age of animals [5,14], artificial insemination (AI) [15], and natural mating [13]. Nonetheless, usual epidemiologic analytic frameworks like logistic regression are often limited for the analysis of high-dimensional, imbalanced and nonlinear data, and may be poorly adapted to epidemiological datasets with a large number of predictor variables (parameters) in relation to the number of observations given the high susceptibility to overfitting [16,17].

Feature selection methods provided by machine learning (ML) approaches are an interesting, flexible and robust alternative for identifying predictors that contribute to disease occurrence. Among these, the random forest (RF) algorithm [18] has been regarded as one of the most precise prediction methods, having advantages such as ability to determine variable importance, ability to model complex interactions among independent variables, and flexibility to perform several types of statistical data analysis, including regression, classification and unsupervised learning [19]. Briefly, RF builds a collection of decision trees based on randomly and independently selected subsets of data, and a simple majority vote among all trees in the forest is taken for class prediction. A clear difference from traditional statistical frameworks is that RF performs a data-driven analysis without making a priori assumptions about the structure of data or the relationship between the response and independent variables, and is less sensitive to spatial autocorrelation and multicollinearity issues [17,20]. Its high predictive power has been supported by previous comparative studies with other ML methods [21-25].

The use of RF allows for a new way of modeling and extracting information from observational data, thus contributing to a better understanding of a target system and mechanism that are, in general, complex and nonlinear. However, according to the authors’ knowledge, there are a limited number of studies in veterinary epidemiology that adopt ML-based methods, and most of them still neglect the importance of proper and careful tuning of models parameters [26-28]. For example, RF was used in a cross-sectional study that aimed at assessing risk factors that may have led to spillover of pH1N1 from humans to swine in Cameroon, Central Africa [26]. In human epidemiology, RF has been already applied in Diabetic Retinopathy (DR) classification analysis for early detection of this illness based on clinical and fundus photography data [16]. Results suggested that RF was a valuable tool to diagnose DR, producing higher classification accuracy than logistic regression, and that the most relevant variables detected by this ML algorithm are meaningful and correlate well with known risk factors.

In this paper, we aim to investigate the use of RF in the analysis of cross-sectional data collected in a BVDV prevalence study. As previously discussed, the application of this ML algorithm is still uncommon for this type of task. Hence, this study has the following main objectives: (1) train a RF model that provides a good predictive power for the collected data, (2) perform a variable importance analysis using the RF model and the well-established Gini index method to identify potential BVDV predictors, (3) investigate the effect of feature selection on the overall performance of the RF model, carefully assessing the impact on the accuracy and the sensitivity-specificity balance, and, finally, (4) compare RF performance with that obtained by other popular ML algorithms and by logistic regression, examining their predictive power and robustness on the scenario of interest.

## Materials and methods

Based on data collected from a prevalence study of reproductive disease in dairy cattle in the State of Rio Grande do Sul, Brazil, a RF model was trained and evaluated with respect to model accuracy, followed by variable importance analysis. All procedures performed for this study was approved by the Institutional Animal Care and Use Committee (Federal University of Rio Grande do Sul, project number: 28288, Porto Alegre, Brazil).

### Study design-data collection

#### Study area and target population

Rio Grande do Sul is the southernmost state of Brazil, with a total area of 268 781.896 km^2^ and 497 municipalities. The cattle population is approximately 13.5 million, 10% of which are dairy cattle [29]. Rio Grande do Sul is the second largest milk-producing state, in which milk production is clustered in six well-defined regions [30]. The study area is explained in more detail in [31].

The target population of data collection included all dairy herds in the state of Rio Grande do Sul. According to the official data from the Office of Agriculture, Livestock and Agribusiness of the State of Rio Grande do Sul 81 307 dairy herds were registered. Descriptive statistics of the studied population can be found in Additional file 1.

#### Survey design and sample collection

First, a cross-sectional survey was performed to estimate the BVDV, *Neospora caninum* and Infectious Bovine Rhinotracheitis (IBR) prevalence in dairy herds based on (bulk tank milk) BTM samples and to identify the associated risk factors, required by the Office of Agriculture, Livestock and Agribusiness of the State of Rio Grande do Sul. A one-stage stratified random sample design was used. Those farms from which one BTM sample was collected were considered a sampling unit. A stratified sample, which was proportional to the herd population present in each of the seven regions, was performed, and each herd was randomly sampled from all the individual strata. These regions are subdivisions of Brazilian states that are grouped according to proximity and share common agroecological characteristics. The sample size was calculated using R Foundation for Statistical Computing, Vienna, Austria (Package EpiCalc), considering the following parameters: total dairy herds registered at the moment (81 307), 50% expected prevalence, 95% confidence interval, and 5% of absolute precision. The minimum sample size required was 384 dairy herds; however, 388 herds were collected to have a safety margin of extra farm samples.

#### Bulk tank milk collection

For each herd, a total of 12 mL of milk was collected directly from the milk container immediately after the entire volume had been homogenized. During sampling and transportation, the raw milk was kept under refrigeration between 2 and 8 °C without preservatives. Following an overnight rest, a 1.2 mL sample of skim milk was collected and kept at −20 °C until analysis.

#### Serological assay and interpretation

The SVANOVIR BVDV p80-AB blocking BVDV ELISA (enzyme-linked immunosorbent assay) was used to detect the BTM samples positive for anti-BVDV antibodies. This blocking ELISA was developed to identify antibodies against the protein p80/NS3, which enables the differentiation between vaccination antibodies and antibodies produced by natural infection. All milk samples were centrifuged for 15 min at 2000 × *g*, according to the manufacturer’s instructions. The absorbance at a single wavelength of 450 nm (A_450_) was determined using a spectrophotometer (Asys Expert Plus, Asys Hitech GmbH, Austria). For the herd prevalence, the percent of inhibition (PI) values were calculated in the same manner as the positive control, as well as for each sample, using the following formula:

1 $$ PI=\frac{O{D}_{Negative\  control}-O{D}_{Sample\  or\  Positive\  control}}{O{D}_{Negative\  control}}\times 100 $$

Herds with PI ≥ 30% were considered to have a high probability to harboring an active infection and/or to have at least one positive cow contributing to the sample.

### Random forest

**Figure 1:**
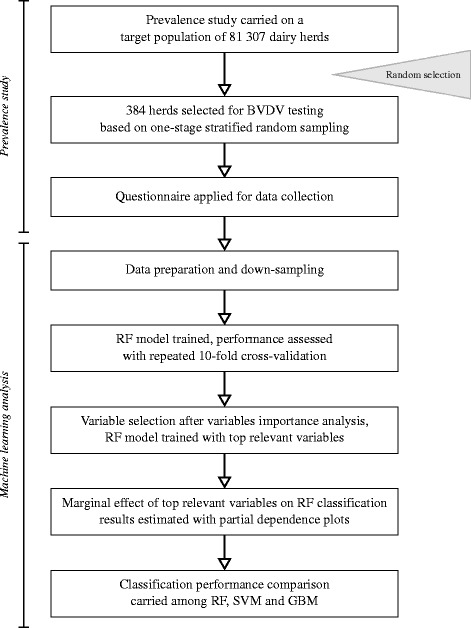
**Flowchart of the study design.** Representation of each step of the study.

In this study we built a RF classifier based on the epidemiological observational data collected from a set of BVDV positive (24%) and negative (76%) farms. The model training process is represented in the flowchart of the study (Figure 1). Since RF algorithm is not routinely used in veterinary epidemiology, we dedicate this section to explain its basis.

**Figure 2:**
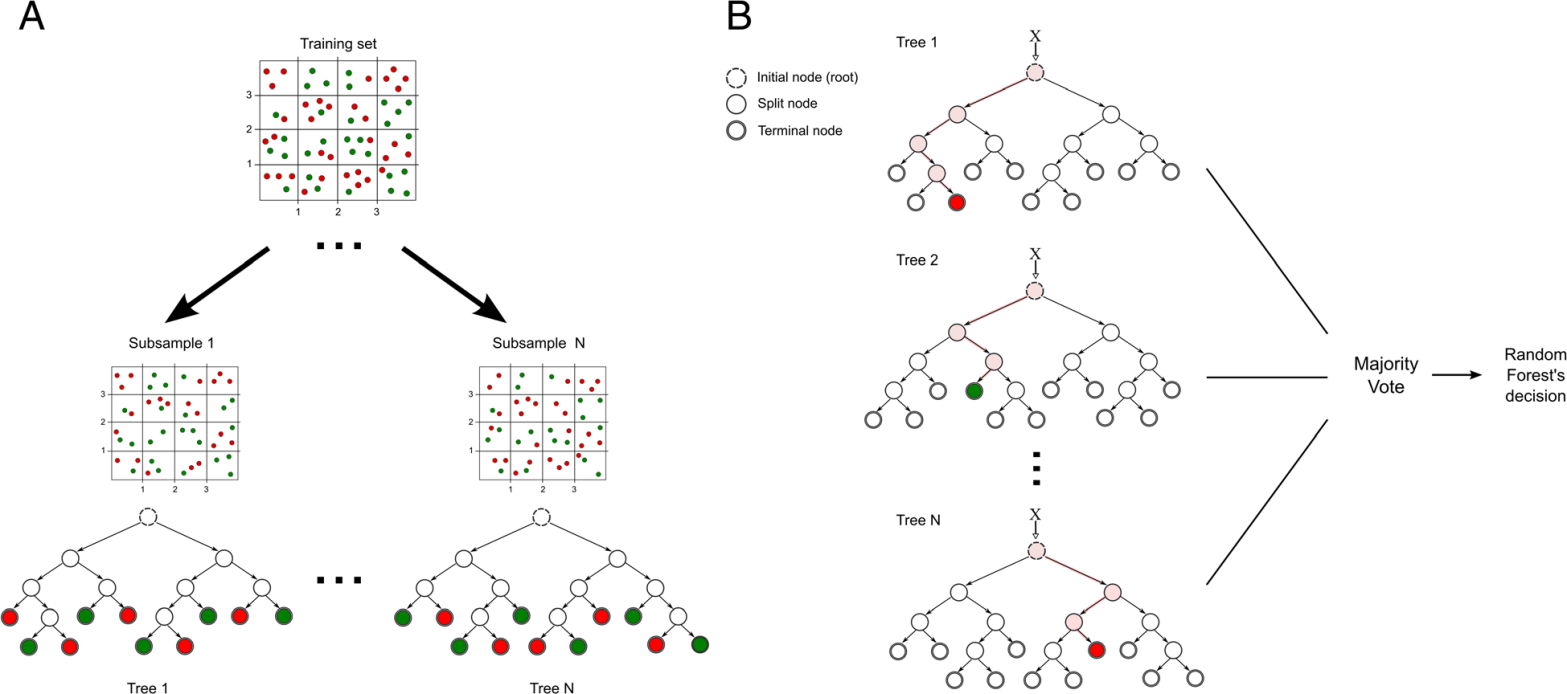
**Random forest model.** Example of training and classification processes using random forest. **A)** Each decision tree in the ensemble is built upon a random bootstrap sample of the original data, which contains positive (green labels) and negative (red labels) examples. **B)** Class prediction for new instances using a random forest model is based on a majority voting procedure among all individual trees. The procedure carried out for each tree is as follows: for each new data point (i.e., X), the algorithm starts at the root node of a decision tree and traverse down the tree (highlighted branches) testing the variables values in each of the visited split nodes (pale pink nodes), according to each it selects the next branch to follow. This process is repeated until a leaf node is reached, which assigns a class to this instance: green nodes predict for the positive class, red nodes predict for the negative class. At the end of the process, each tree casts a vote for the preferred class label, and the mode of the outputs is chosen as the final prediction.

Random forest is an example of a machine learning method for classification and regression analysis that uses an ensemble of randomized decision trees to define its output. The algorithm constructs a collection of decision trees using the traditional classification and regression trees methodology (CART) [32] (Figure 2A) and combines the predictions from all trees as its final output when predicting the class of new instances (Figure 2B), making it accurate and robust in relation to other ML algorithms [18]. In classification tasks, as is the case in the current study, combination is performed by means of majority voting among the individual decision trees. Briefly, when classifying new instances from an input variables vector, the mode of the classes returned by the classification performed by individual trees is defined as the final output of the RF model. Hence, supposing we have 100 trees in the forest, among which 70 predict a particular instance as positive for BVDV and the other 30 predict it as negative, the final RF prediction would be positive for BVDV given the majority of votes for this class.

Each decision tree composing the forest has the standard flowchart-like structure, in which internal (split) nodes test variables and branch out according to their possible values, and leaf (terminal) nodes assign a classification for all instances that reach the leaf. The tree growing process in RF is also based in binary recursive splitting that aims at maximizing the decrease of impurity at each node, where impurity can be evaluated by heterogeneity for classification trees (if the response is of categorical type). Nonetheless, in constructing the ensemble of trees, RF incorporates two types of randomness. First, each tree is built using a random bootstrap sample of the original training data (~2/3 of samples), drawn by sampling with replacement (Figure 2A). Second, at each candidate split in the tree growing process, a subset of variables is randomly selected among all available variables to decide node splitting, and the best split among these variables is chosen based on the smallest node impurity [18,33]. Here, we adopt the well-known Gini index as a measure of node impurity. The tree growing procedure is performed recursively until a minimum node size is reached, which is parameterized by the user, or until no further improvement can be made [34]. The two main parameters of the RF algorithms are the number of random variables (predictors) to evaluate at each node split and the number of trees to grow in the ensemble.

The methodology underlying the RF algorithm has interesting properties that make it especially appealing for classification tasks. To begin with, the mechanism applied for tree growing allows the estimation of the most important variables for classification, and generates an internal unbiased estimate of the generalized error drawn from the data left out of the bootstrap sample used as a training set, called out-of-bag (OOB) data, which corresponds to about ~1/3 of the original data. In addition, the fact that the predicted class represents the mode of the outputs returned by individual trees gives robustness to this ensemble classifier in relation to a single tree. Finally, the bootstrapping procedure and the out-of-bag estimates make RF more accurate and less sensitive to issues such as overfitting, outliers and confounding in comparison to other statistical and machine learning methods [18,33].

In this study, the learning process was carried out with the randomForest and caret packages for the R statistical environment [35,36].

### Data preparation

Given the severe class imbalance observed in the data and the general difficulty of machine learning methods to handle this issue [37], we have incorporated a down-sampling procedure in the model learning functions provided by the caret R package, which samples the majority class to make its frequency closer to the rarest class. This procedure aims at avoiding the ML algorithm’s tendency to be strongly biased towards the majority class, consequently misclassifying a lot of instances related to the minority class.

The original dataset was randomly and uniformly (i.e., maintaining the same proportion of classes as in the original dataset) split into a training set (80% of observations) and an independent testing set (20% of observations). This subdivision reflects an attempt to compose a minimum sample size that would be representative in future applications of the model and is a common strategy for evaluating ML models when external validation data is not available. The training set was applied for training our classifier using a cross-validation process and the testing set was further used to compare models performance based on independent test data.

### Variables

The set of 40-predictor variables collected in the survey performed and used to train the BVDV classification model were: (1) who inseminates the animals, (2) number of neighboring farms that have cattle, (3) what proportion of the farm income is based on milk production, (4) for how many years has the farm produced milk, (5) frequency of technical assistance, (6) is rectal palpation performed routinely, (7) the number of different inseminators in the last year, (8) what is the origin of the bulls, (9) frequency of veterinary assistance, (10) are the animals placed in quarantine before introduction, (11) what is the origin of animals brought into the farm, (12) how often does the fence between/among farms that hold cattle collapse, (13) how the cows are milked, (14) was there an increase in abortions, (15) does calving occur in closed barns, (16) number of cows lactating at the sampling moment, (17) were animals vaccinated for BVDV, (18) was there a rise of mating failure, (19) do animals share the same feed and water containers, (20) number of cows not lactating at the sampling moment, (21) is colostrum stock available, (22) total farm area in hectares, (23) are paddocks available for sick animals, (24) who administers the medications, (25) is blood from a sick animal injected into the healthy ones (“Premunição”), (26) within the last year have animals been sent to fairs, (27) has the farmer seen weak born calves, (28) were pregnant cows introduced, (29) total area for cow farming, (30) has the farmer seen weak calves, (31) were new animals introduced in the last year, (32) possibility of direct contact (over the fence) between animals from the neighboring farm, (33) animals are grouped based in age category, (34) is the inseminator always the same, (35) does the farm have technical assistance, (36) is natural mating used, (37) does the farm have bulk milk tank, (38) is artificial insemination used, (39) does calving occurs in the fields, (40) does the farm have veterinary assistance. See Additional file 2 for the frequency of important predictor variables.

### Model training

The RF model was trained with the training set derived from the original data (i.e., 80% of data) and the complete set of variables using the randomForest package in R. The number of trees induced in the training process was configured to 500 trees following the suggestion of the authors, and the number of variables (*mtry*) randomly sampled as candidates for node splitting during the tree growing process was optimized using the caret package in the R environment. In training the model, we adopted a repeated 10-fold cross-validation technique to better estimate its performance and generalization power, and to prevent overfitting and artificial accuracy improvement due to use of the same data for training and testing the classifier.

Once the model was trained, we investigated the effect of multicollinearity over the performance of RF. For this purpose, we computed the correlation matrix for the set of 40 variables using Pearson correlation and identified highly correlated predictors among our independent variables. Next, we selected some of the highly correlated variables to discard from the analysis based on plausibility criteria and repeated the RF training process without these variables, comparing its performance with the original RF model.

An interesting property of RF is that it naturally provides estimates of variable importance, which are computed during model training by evaluating the average decrease in the nodes’ impurity measured by Gini index. The importance of a variable is defined as the Gini index reduction for the variable summed over all nodes for each tree in the forest, normalized by the number of trees [38]. Hence, the higher the Gini importance, the more relevant that variable is for maintaining the predictive power of the RF model. Although RF are capable of modeling a large number of variables and achieving good prediction performance, finding a small number of variables with equivalent or better prediction ability is highly desired not only because it is helpful for interpretation, but also easy for practical use as strategies for disease control [38].

Thus, after running the first round of model training and obtaining the Gini importance for each of the 40-predictor variables of our data set, we performed a restricted forward feature selection and verified the impact of variables inclusion over the model’s predictive accuracy in an incremental fashion. This step aims at identifying irrelevant variables that may mislead the algorithm and increase the generalization error [39]. Specifically, we trained several RF models, starting from a model trained upon a single variable, and subsequently adding new variable one at a time, from the most relevant to the least relevant. For each of the classifiers generated, we evaluated its performance by computing the AUC score, specificity and sensitivity for the OOB data. Based on this analysis, we selected the top important predictor variables that optimized model’s performance and ran the training process again, generating a simplified RF classifier that considers only the most impactful variables.

Finally, we explored the relevance of variables for classification results by partial dependence plots, which are useful for providing insights of the marginal effect of a given variable over the desired outcome. The partial dependence of a variable’s effect is best understood by examining general patterns in relation to the values of the predictor variable rather than the specific values of partial dependence [40]. Because we are modeling binary classification (i.e., presence/absence of BVDV), partial dependence values are given in “logit” scale and are computed in relation to the probability for the positive class [19].

### Model performance assessment

The model performance was assessed by computing the total prediction accuracy (ACC), specificity (SPE) and sensitivity (SEN) based on the confusion matrix. This matrix quantifies the number of instances in the test data classified as false positive (FP), true positive (TP), false negative (FN), and true negative (TN). We also plotted the area under the Receiver Operating Characteristic (ROC) curve. The area under the ROC curve gives us the AUC score, interpreted as the probability that a classifier will rank a random chosen positive instance higher than a random negative one.

2 $$ ACC=\frac{TP+TN}{TP+TN+FP+FN} $$

3 $$ SPE=\frac{TN}{TN+FP}\times 100\% $$

4 $$ SEN=\frac{TP}{TP+FN}\times 100\% $$

### Comparing RF to other machine learning methods

In order to assess the predictive power of RF in comparison to other ML techniques, we performed a comparative evaluation of the RF classifier with two other popular methods, namely Support Vector Machine (SVM) and Gradient Boosting Machine (GBM), which have also not been extensively assessed in veterinary epidemiology. SVM was introduced by [41] and is based on a statistical-learning technique known as structural risk minimization [41,42], being first used in observational epidemiology studies in 2005 [43]. GBM, on the other hand, is an ensemble method that combines regression trees with weak individual predictive performances into a single model with high performance [34,40].

For such comparison, we adopted the same procedure used for RF training, i.e., 10 repetitions of 10-fold cross-validation, assuring that the exact same data points are used in each step of model training and testing. In other words, we maintained the same subsampling of the training data used in the cross-validation process. In addition, we applied the caret R package to train SVM and GBM models, tuning some of the parameters involved in order to carry a fair comparison with RF. Based on the results from cross-validation, we performed a first round of comparison among models, contrasting their AUC score, sensitivity and specificity drawn from the average confusion matrix. Finally, the differences between models performance in terms of AUC scores were assessed with a pairwise Wilcoxon rank test in order to test for statistical significance.

### Comparing RF to logistic regression

Since we are interested in suggesting the use of RF as an alternative method for traditional statistical approaches, we also assessed its performance relative to logistic regression, which is frequently used for the analysis of risk factors. Logistic regression was estimated with the glm() function in R environment and performance evaluation was carried out based on 10 repetitions of 10-fold cross-validation using the caret R package. To assure a fair comparison, we run the logistic regression analysis with the same distribution of data used for RF training among folds and across all repetitions of cross-validation.

### Models evaluation on independent testing data

In addition to evaluating the methods performance using cross-validation, we also assessed their predictive accuracy with an independent test set derived from the original data. As aforementioned, during data preparation the original data set was subdivided in training data (80%) and testing data (20%), which is not used in the cross-validation procedure and thus can be regarded as an independent test set.

This approach is recommended when no external independent data are naturally available [44,45], which is the case in our study. Although cross-validation is well known for providing precise and unbiased estimative of the predictive accuracy and generalization power of ML classifiers, we opted to follow the common practice and conduct another comparison among models with explicitly independent data.

## Results

### Performance of the RF model

The confusion matrix for the tuned RF model trained with all available predictor variables (*n* = 40) and *mtry* = 25 (optimized value computed by caret R package), averaged over the 10 repetitions of the 10-fold cross-validation, is shown in Table 1. We evaluated the confusion matrix for the final RF model, obtaining the following performance metrics: ACC: 67.42% (±3.69); SPE: 67.65% (±3.85) and SEN: 62.26% (±3.44). Despite optimizing parameters and adopting a down-sampling procedure, RF had an average error rate of 32.03% for the negative class (negative for BVDV) and 36.78% for the positive class (positive for BVDV), with a standard deviation of 1.30% and 2.46%, respectively.

**Table 1:** **Classification performance of RF model for the 40 variables. Confusion matrix for the RF model trained with the complete set of predictor variables (** ***n*** **= 40) and a down-sampling procedure, estimated by averaging the results over ten repetitions of 10-fold cross-validation. Standard deviations are given in parenthesis** ^*^

		**Real**	
		BVDV-negative	BVDV-positive
**Predicted**	BVDV-negative	114.0 (6.5)	2.83 (0.25)
	BVDV-positive	54.5 (6.5)	4.67 (0.25)

^*^Performance metrics: ACC: 67.42 (Sd. 3.69); SPE: 67.65 (Sd. 3.85) and SEN: 62.26 (Sd. 3.44).

Analysis of the correlation matrix computed for the set of 40 variables (Additional file 3) suggested that a small set of independent variables is highly correlated. Based on plausibility criteria, we eliminated the highly correlated variables, namely (5) frequency of technical assistance, (9) frequency of veterinary assistance, (11) what is the origin of the animals brought into the farm and (30) has the farmer seen weak calves, and repeated the training process. We observed a minimal change in the RF model performance after the elimination of correlated variables, with the highest (but still modest) impact found for sensitivity, i.e., an increase from 62.26% to 65.10%.

### Variable importance

**Figure 3:**
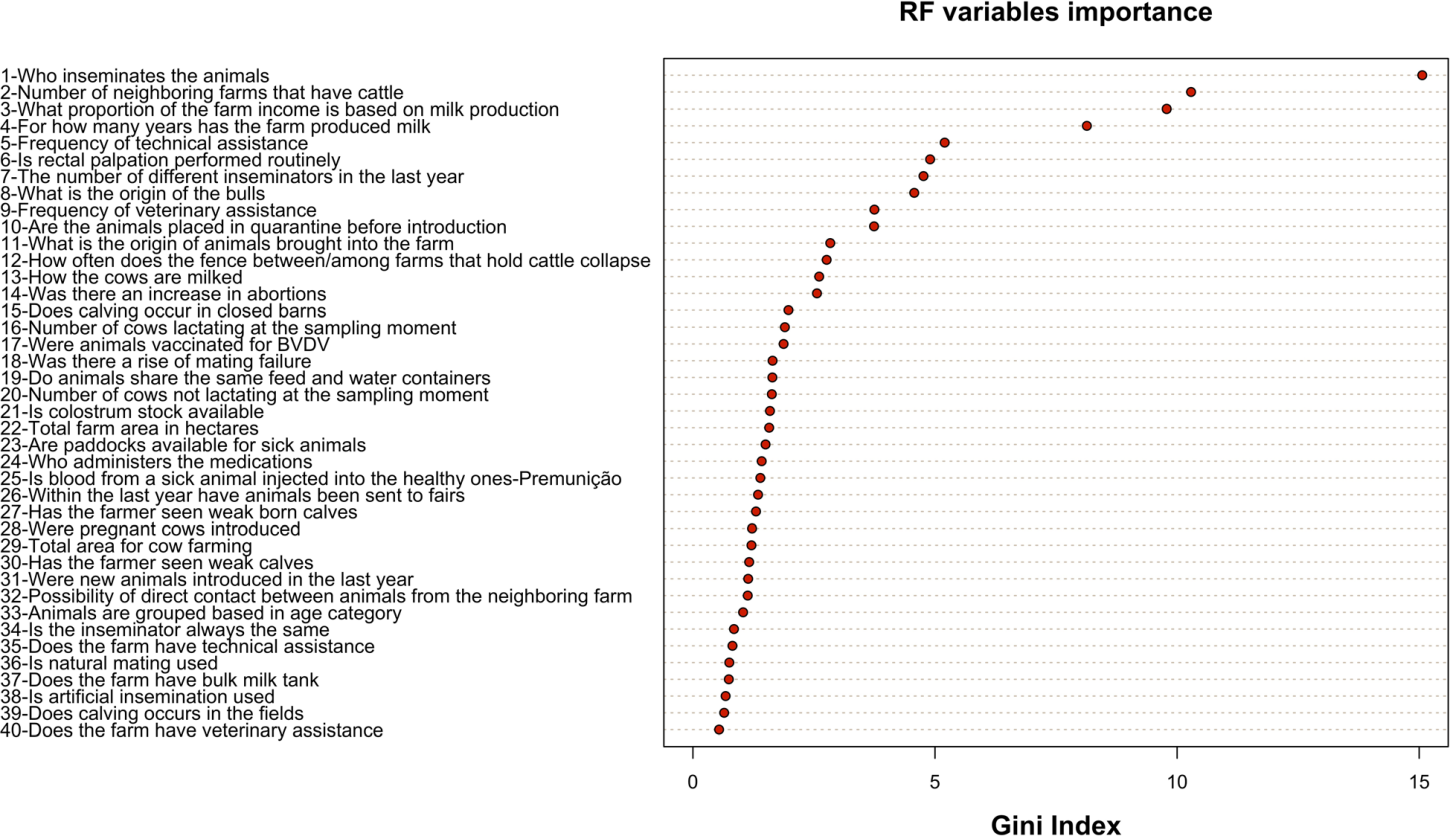
**Variable importance analysis performed by RF.** The set of 40 variables used for classification, ordered by their importance as estimated by the RF model.

We performed a variable importance analysis assessing the average decrease in the nodes’ impurity measured by the Gini index during the construction of the random forest model. Figure 3 presents the result of this analysis, with the variables ranked by their Gini importance. As we may observe, the variables (1) who inseminates the animals, (2) the number of neighboring farms that have cattle, (3) what proportion of the farm income is based on milk production and (4) for how many years has the farm produced milk are the four most important variables for BVDV prediction found in this analysis, since they are associated to the highest Gini importance.

**Figure 4:**
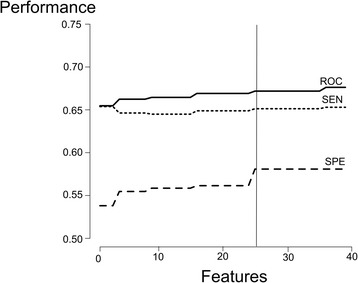
**Result of restricted forward feature selection.** Performance of the RF model evaluated by means of a restricted forward feature selection. Several RF classifiers were trained adding each of the predictor variables at a time, following the rank obtained from the variable importance analysis, which is based on the mean decrease of Gini index.

The result of the restricted forward feature selection carried after variable importance analysis can be seen in Figure 4. The best performance balance considering AUC score, specificity and sensitivity, as well as model complexity, seems to be associated with the model trained with the top 25-predictor variables. Hence, the RF training procedure was repeated for this subset of variables (Figure 3), optimizing model’s parameters by means of the caret package in R. The best tune for *mtry* was 16, and the classification results for this model are shown in the confusion matrix depicted in Table 2. We noticed that the model trained with 25 variables, generated after feature selection, presented a slight increase in the average accuracy (ACC: 67.75%) and specificity (SPE: 67.98%) in relation to the model trained with the total set of variables, whilst no variation was observed for sensitivity. Nonetheless, this increase is not statistically significant, and hence in this scenario feature selection does not seem to introduce important benefits to the performance of the RF model.

**Table 2:** **Classification performance of RF model for the top 25 variables. Confusion matrix for the RF model trained with the top 25-predictor variables selected after variable importance analysis, estimated by averaging the results over ten repetitions of 10-fold cross-validation. Standard deviations are given in parenthesis** ^*^

		**Real**	
		BVDV-negative	BVDV-positive
**Predicted**	BVDV-negative	114.55 (6.8)	2.8 (0.20)
	BVDV-positive	53.95 (6.8)	4.7 (0.20)

^*^Performance metrics: ACC: 67.75 (Sd. 3.69); SPE: 67.98 (Sd. 3.85) and SEN: 62.26 (Sd. 3.33).

**Figure 5:**
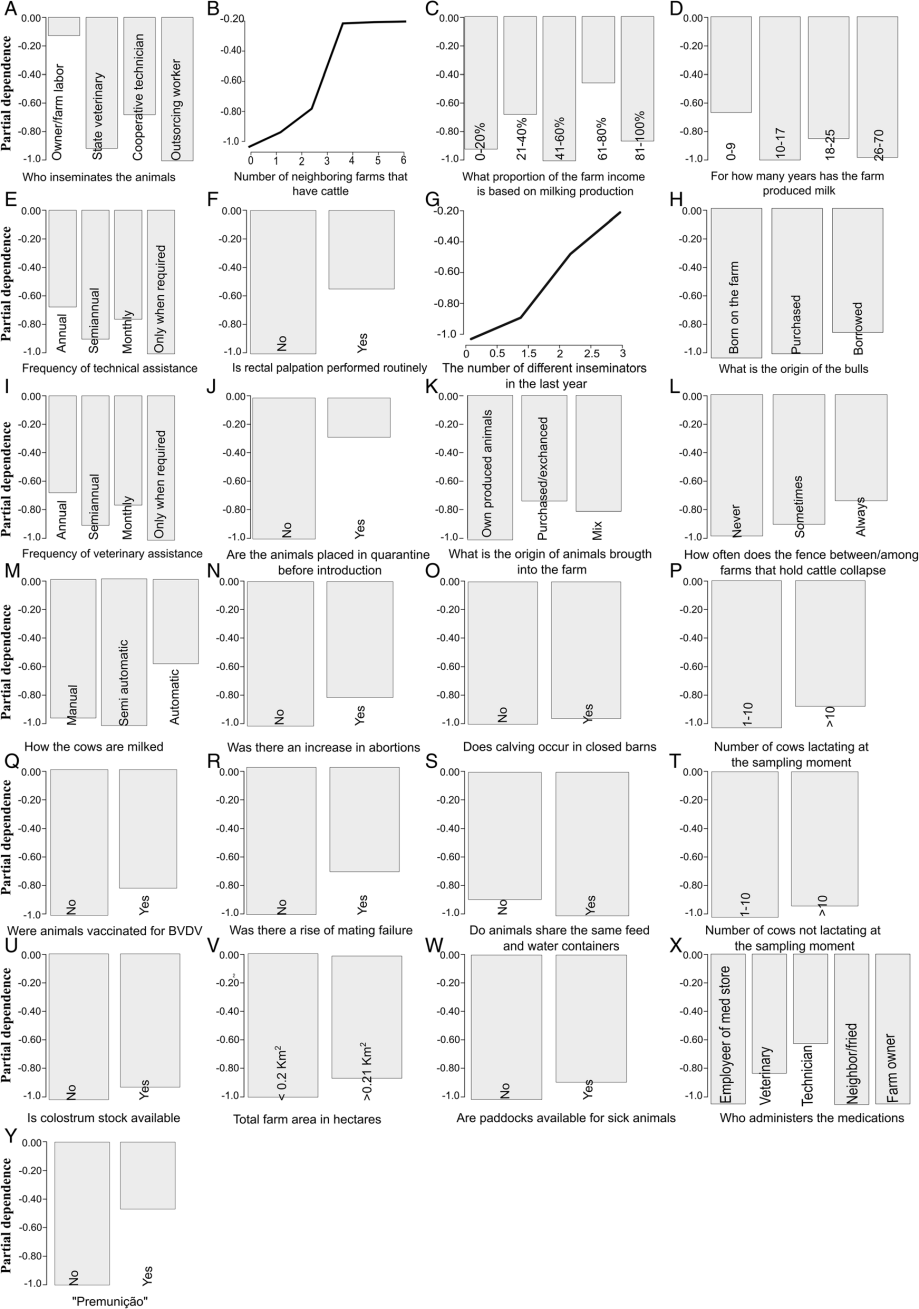
**Partial dependence plots for the top 25 variables.** Partial dependence plots for the top 25 variables with the variable importance scores as calculated by random forests. Plots show the partial dependence of a Relative Occurrence Index value for BVDV on each predictor variable; the y-axis is given in log scale [the logit function gives the log-odds, or the logarithm of the odds p/(1 − p)].

To better understand the effects of the most important variables over classification results, we explored the partial dependence plots for the top 25-predictors (Figure 5), which give a graphical depiction of the marginal effect of a variable on the class probability. Greater y-values indicate that an observation for a specific variable is associated with higher probability for classifying new instances as BVDV positive.

As this analysis suggests, (B) the number of neighboring farms that have cattle and (G) the number of different inseminators in the last year had a strong linear correlation with BVDV. Moreover, we observed that disease occurrence was highly influenced by observations related to some specific variables, mainly by (A) insemination performed by the owner or farmer, (C) milk production representing about 61-80% of far income, (E) technical assistance conducted annually, (F) rectal palpation performed routinely, (I) veterinary assistance held annually, (J) animals placed in quarantine before introduction, (M) milking process performed in an automatic fashion, (X) administration of medications performed by a technician and (Y) the regional habit of injecting blood from a sick animal into a healthy one (“Premunição”). In contrast, there was no significant relationship between BVDV occurrence and the variables (O) does calving occurs in closed barns, (P) number of cows lactating at the sampling moment, (S) do animals share the same feed and water containers, (T) number of cows not lactating at the sampling moment, (U) is colostrum stock available and (W) are paddocks available for sick animals.

### Comparative evaluation of RF, SVM and GBM

The results of the comparative analysis based on the average AUC scores, computed as the mean of the area under the ROC curves over all repetitions of cross-validation, were 0.702 for RF, 0.690 for GBM and 0.687 for SVM. The highest specificity was achieved by SVM (69.45% ± 4.05), followed by RF (67.65% ± 3.85) and GBM (66.15% ± 2.58). On the other hand, RF achieved the highest sensitivity (62.26% ± 3.44), followed by GBM (61.73% ± 5.33) and SVM (57.60% ± 4.73).

**Figure 6:**
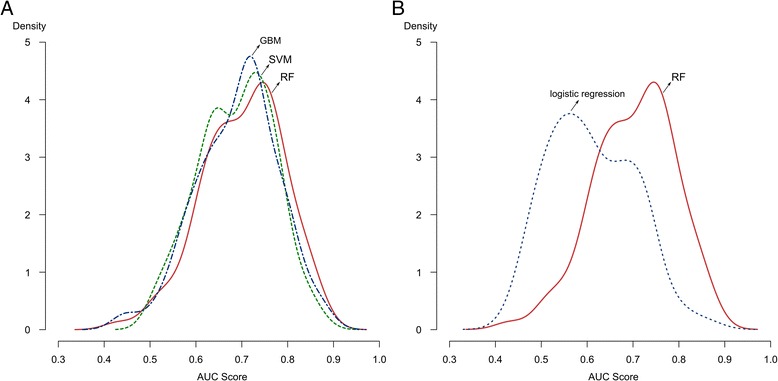
**Comparative evaluation of RF against GBM, SVM and logistic regression based on repeated cross-validation.** The performance of the models over several resamples are summarized by a kernel density estimator, which indicates a narrow distribution and slightly shifted to the right (higher values) for RF **A)** in relation to SVM and GBM and **B)** in relation to logistic regression.

In a visual analysis of density distributions of AUC scores obtained for each classifier (Figure 6A), RF presents a distribution slightly shifted to the right in relation to others, indicating a tendency in provide a better predictive accuracy than GBM and SVM. Nonetheless, differences among methods performance in terms of AUC scores are not statistically significant according to a pairwise Wilcoxon Ranked Sum test using the Benjamini-Hochberg procedure to correct for multiple comparisons. The lowest *p*-value was associated to the comparison between RF and SVM (*P*-value = 0.064), followed by the comparison between RF and GBM (*P*-value = 0.075).

We also compared the distribution of sensitivity and specificity metrics across all repetitions of cross-validation following the same methodology, and we found that SVM has better specificity performance than RF and GBM (*P*-value < 0.05), while both RF and GBM outperform SVM in terms of sensitivity (*P*-value < 0.05).

### Comparison between RF and logistic regression

As expected according to our theoretical motivation, we observed a superior performance of RF relative to logistic regression. While RF had an average AUC score of 0.702, the model estimated by logistic regression achieved an AUC score of 0.610 across all repetitions of cross-validation. The density plots drawn from the cross-validation procedure makes evident the better predictive power of RF, which presents an AUC scores distribution shifted to the right of that related to logistic regression (Figure 6B).

Moreover, we observed that the classification provided by RF is much more balanced in terms of sensitivity and specificity than logistic regression. The average specificity was 67.65% (±3.85) for RF and 61.36% (±3.33) for logistic regression, while the average sensitivity achieved by these methods were 62.26% (±3.44) and 56.30% (±3.84) for RF and logistic regression, respectively.

### Models evaluation with independent testing data

**Figure 7:**
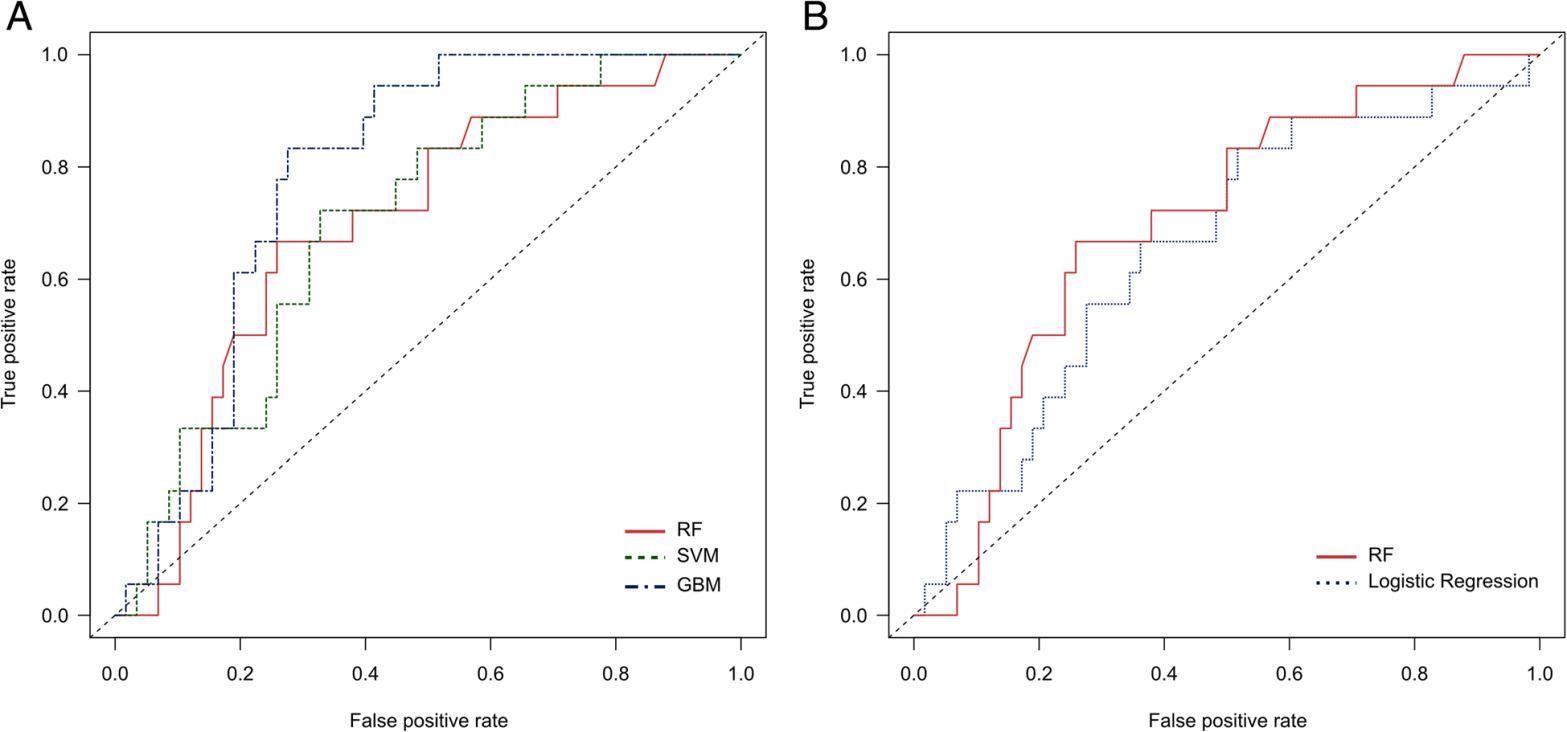
**Evaluation of models performance for independent test data. A)** RF, SVM and GBM were also compared using an independent test set, which corresponds to the 20% portion of data that was not used in the training and cross-validation procedures. According to the ROC curves, the GBM classifier outperforms RF and SVM. **B)** Relative to the logistic regression, a traditional statistical approach used for the analysis of risk factors, RF achieved a more robust performance.

In addition to the comparative analysis carried out among classifiers using cross-validation, we evaluated the models’ predictive accuracy with independent test data. Results in terms of the ROC curves are shown in Figure 7A for the ML algorithms. The corresponding AUC scores are 0.697 for RF, 0.703 for SVM and 0.785 for GBM.

Differently from the cross-validation technique that ensures every instance in the data set will be used exactly once for model validation, the initial partitioning of data is performed a single time in a random fashion, and may generate a testing data set for which GBM, fortunately, have a superior performance – an effect that is out of our control. To test for this possibility, we repeated the process of model training and testing 10 times, each of which with a random (and thus potentially different) partitioning of data into training and testing sets, keeping the proportions of 80% and 20%, respectively. We performed this procedure for the three classifiers, i.e., RF, SVM and GBM, and compared their average performance for the independent test data across all repetitions. We observed that RF outperforms the other classifiers in 6 out of the 10 repetitions, while in the remaining 4 the best performance is achieved by GBM (Additional file 4). Although the average AUC score of RF is only slightly better than GBM, 0.7466 vs. 0.7301, the worst and best performances achieved by RF show a performance gain of 12.09% and 7.13% in relation to the worst and best models trained by GBM, respectively.

Regarding the comparative evaluation between RF and logistic regression, similarly to what was observed from the cross-validation procedure, RF presented a more robust performance for independent testing data in relation to logistic regression. The ROC curves are shown in Figure 7B, corroborating the better predictive accuracy of RF in contrast to logistic regression.

## Discussion

In this study, we trained a RF model based on cross-sectional data derived from an investigation for BVDV prevalence carried in Southern Brazil, aiming to identify important predictors for disease occurrence and to evaluate the predictive power of this machine learning model in this specific domain. To the best of our knowledge, this is one of the few studies in veterinary epidemiology that performs an investigation based on machine learning algorithms adopting a careful training process, which encompasses parameters optimization and a strategy to treat a severe class imbalance problem. In addition, it was also the first time that a comparative evaluation among RF, SVM and GBM models was held in this context, adopting appropriate methods for model tuning and a repeated 10-fold cross-validation technique.

Based on the classification results by RF, we noticed that our model’s performance has shown an overall good predictive accuracy and quite balanced sensitivity and specificity across all repetitions of the cross-validation. The data-driven analysis carried by RF, without a priori assumptions about the relationship between the dependent and independent variables, has a great potential to outperform the traditional logistic regression, as experimentally verified for our data, suggesting that RF could be a valuable tool in cross-sectional studies. The reader should be aware that our results do not come from basic measures of total classification accuracy and error rates; instead, we have adopted robust evaluation approaches and made important interventions for training and optimizing the machine learning classifiers, providing a more appropriate application of these methods to our scenario. Specifically, we have optimized the number of predictor variables selected for splitting a new node during the production of the decision trees, and we decided to not optimize the number of trees in the forest based on the former discussion that RF is not very sensitive to this last parameter [35].

Despite its satisfactory performance, our classifier has missed on average more positive than negative cases of BVDV, even after the application of the down-sampling strategy (Table 1). Most standard algorithms assume or expect balanced class distribution or equal misclassification costs [46], so when a complex imbalanced data set is used, these algorithms fail to properly represent the distributive characteristics of the data and resultantly provide unfavorable accuracies across the classes of the data [46]. In our data we found an imbalance in a form commonly referred to as “intrinsic”, which means the imbalance is a direct result of the nature of the data space [46]. We analyzed the effects of the down-sampling procedure over classifiers performance, comparing the results obtained from training with and without handling the data imbalance issue, and we observed that all three methods suffered impact from the severe data imbalance over their sensitivity. When training is carried without treating this issue, models’ sensitivities were in the approximate range of 11.5% to 20%, which is clearly lower than the values of 57.60%, 61.73% and 62.26% achieved by SVM, GBM and RF, respectively. Hence, we observed that adopting this pre-processing strategy in data sets containing classes that are highly under-represented in comparison to others may introduce important benefits for data analysis, although in this case it did not completely solved this issue.

The final variables ranking in a descending order of importance as provided by RF’s variable importance analysis (Figure 3) suggests that the main variables involved in BVDV prediction are related to reproduction-associated factors, movement of many people into and out of the farms, and direct contact among animals, as we discuss further. Feature selection has been previously shown to result in slight error reductions [47], and this step is normally performed in order to remove variables that do not contribute to the performance of the model, either because they do not play an important role on error reduction or because they have a minimal effect on the discriminant power of the RF classifier [48]. One can notice that although performance improvement was not so expressive after feature selection (Table 2), we still observed a slight gain in terms of accuracy and specificity. The top 25 variables model is therefore more efficient, as it provides a performance as good as the model trained with the complete set of 40 variables despite the reduction in model complexity.

Regarding the results of variable importance analysis, we discuss only the most relevant variables due to space limitation. The most impactful variable for BVDV prediction was related to farms that perform AI (Figure 5A), a factor that has been considered a predictor for BVDV globally, especially when semen is used from untested bulls or when farms use AI along with natural mating in order to “guarantee” the success of a pregnancy, a common and unsafe practice in Brazil [10]. AI is an important route of transmission of BVDV because semen remains infective, which is evident by the demonstration that susceptible cows can become infected following insemination [15,49-51]. A remarkable new association that we found was that when AI is performed by the owner or someone that is responsible for the farm, a common reproductive practice in Brazil and other countries, the influence on BVDV cases was evidently harmful, increasing the probability of disease occurrence. It was also reaffirmed that the number of neighboring cattle farms where there is chances of direct contact between cattle over the fence was a predictor for BVDV [13]. Others have identified the direct contact over fence lines one of the hardest to control [52]. In our analysis, we showed that the partial dependence of BVDV on this variable increases as the numbers of neighbors’ increases, and that BVDV occurrence rises abruptly when there are three neighboring farms. The occurrence of BVDV was also influenced by factors related to milk production. When milk production was reported to represent 61 to 80% of farm income (Figure 5C), we observed a high association with BVDV, most likely due to milk production with intensive pressure on cow performance. It was found that farms that have produced milk for up to nine years had the highest influence on disease occurrence in contrast to farms that have been harvesting milk for longer periods (Figure 5D) this fact may be related to the inexperience of the farmer.

Partial dependence analysis also suggested that rectal palpation performed routinely (Figure 5F) causes significant influence on BVDV occurrence. It has been found that indirect transmission of BVD virus can be spread by veterinary equipment such as nose tongs, needles and protective rubber gloves worn during rectal examination [53,54]. Others [55] had also reported that rectal palpation performed consecutively on different animals without proper hygiene (e.g., without replacing glove between animals) might play an important role in the transmission of BVDV. Moreover, the number of different inseminators that had visited the farm in the past year showed a linear influence on BVDV (Figure 5G). We observed that as the number of inseminators increases, the chances of predicting positive cases of BVDV were also higher, probably due to intense people movement acting as fomites.

In order to compare the RF model against other classifiers that have similar literature, a repeated 10-fold cross-validation was performed, averaging model accuracy measures over all repetitions. We found a better overall performance of RF in relation to SVM and GBM, especially in terms of specificity and sensitivity balance, but results were very close among ensemble-based algorithms (i.e., RF and GBM). Although the difference between the AUC scores of these two classifiers are not statistically significant, we found based on visual analysis of kernel density estimates that the probability distribution of RF is shifted to the right of GBM and SVM distributions, which suggests that RF has a tendency to produce higher AUC scores (i.e., achieve best performance) in relation to the latter. Others had previously found similar results when testing the performance of all tree classifiers, but in the previous study, GBM and SVM performed relatively better than RF [56]. The poor results related to SVM may be due to the fact that the performance and prediction results of this classifier are heavily dependent on the chosen values for the tuning parameters [57-59]. Although we adopted a parameter optimization procedure based on grid search methods that minimize total error rates, a more exhaustive study towards the evaluation of classifier’s performance upon parameters optimization, combined with the application of other optimization techniques, could lead to an even better performance. However, this analysis is out of the scope of our work.

Surprisingly, for tests with independent data, GBM showed an improved performance, which is better and more balanced than the performance achieved by RF and SVM. This may indicate a better generalization power of this algorithm, but it may also be an artifact of data partitioning, which randomly generates a test set for which GBM has a more favorable chance of producing accurate classification. However, due to the random nature of the procedure, repeated partitioning of the original data into training and testing sets may produce results with large variability, both qualitative and quantitative, and consequently provide less consistent insights than the analysis performed with cross-validation. We verified this effect by repeating 10 times the complete training process, from data preparation (and consequently data partitioning) to models evaluation, based on which we observed significant variance in methods performance. Briefly, RF and GBM were always the top-performing classifiers, but in 6 out of the 10 repetitions, RF outperformed GBM, showing that the outcome of this comparison is highly dependent on initial data partitioning. Hence, we emphasize that the 10-fold cross-validation technique is more powerful in reducing overfitting and more precise for assessing the predictive power of machine learning methods, providing an unbiased estimative of how a classifier model will generalize to an independent data set.

It should be noted that GBM is functionally similar to RF because it creates an ensemble of trees and uses randomization during this process. This fact could support the similar results observed for these two methods. However, whereas RF builds the trees in parallel and these trees “vote” simultaneously on the preferred class during prediction, GBM creates a series of trees in which the prediction receives incremental improvement by each tree in the series [60].

In life sciences, random forests have been used to analyze genomic data [61,62], in ecology they have been successfully used as classifiers [19,63,64], and herein they are used for cross-sectional studies in veterinary epidemiology. Random forests proved to have good accuracy, sensitivity and specificity, showing a discriminant power that is highly competitive with other ML-based methods for detecting biologically plausible predictors of BVDV. Based on these results, we believe that RF is a promising computational approach for cross-sectional studies in veterinary epidemiology and should be more frequently considered as an alternative for traditional statistical methods.

Moreover, our model demonstrated a novel use of observational data that goes beyond the previously identified predictors. The application of machine learning extends the usefulness of classical risk factors found on the basis of traditional statistical approaches. Based on the proposed RF model, we could take a closer look at some classical predictors and found important details regarding their relationship with disease occurrence, mainly regarding reproduction management, which should be considered for disease control and eradication. One should take this investigation further ahead in order to clarify how the important reproduction variables contribute to BVDV in other countries.

## Additional files

Additional file 1: Descriptive statistics on the study population. A descriptive analysis has been performed in order to show an overview of the study population. Additional file 2: Frequency of important predictor variables. The prevalence of important predictor variables obtained by serological assay results provides details of disease occurrence in the study population. Additional file 3: Correlation matrix for predictor variables. Negative correlation is represented by red ellipses pending to the left; positive correlation is represented by blue ellipses pending to the right. The exact correlation values are given in the upper panel. Additional file 4: Models performance for 10 randomly generated independent test data sets. The AUC scores are computed for 10 repetitions of model training and testing. In each repetition, a random portion of 80% of data is used for training, and the remaining 20% for testing (independent data).
