# Supplementary material for: What variables are important in predicting bovine viral diarrhea virus? A random forest approach
Source: Vet Res. 2015 Jul 24;46(1):85. doi: 10.1186/s13567-015-0219-7 (PMC4513962; doi:10.1186/s13567-015-0219-7)
Supplement: Additional file 1: Descriptive statistics on the study population. — A descriptive analysis has been performed in order to show an overview of the study population. [file 13567_2015_219_MOESM1_ESM.doc]

**Additional file 1 Descriptive statistics on the study population.**

A questionnaire with both close and open questions (*n* = 40) was applied in 388 farms sampled in this study. We present a general description of the studied farms. The number of people working in the farms was small; with 67.20% reporting that up to two people were involved on dairy activities on daily bases. The average farm size (land) was 33.74 hectares (range: 1-1500). On top of that, 59% of the farm (land) was used for dairy related activity (range: 2-100%). About the experience on dairy production of each farm measured in years, an average of 19.5 years (range: 1-70) was found, and in 25.3% of the families/companies, the milk production represents 41-60% of total farm income. The median number of cows per farm was equal to 3 (range: 1-57), and on the day of the sampling the median of lactating cows was 11.5 (range: 0-130). Moreover, 75% of the farms had up to 21 lactating cows, with median of 3 not lactating cows (Figure below).

Distribution of count number of cows-red dots represents the outliers’ farms.

About the sanitary management, veterinary assistance was reported to be present on 89.7% of the farms and the visits were done predominantly when required by the owner. Most of the owners have participated on agglomeration events, sending animals either for a show, fairs or rodeos. In 53.6% of the farms own only animals born and raised on the own farm, but when an outsourcing animal was introduced, 67.85% reported that the animals were pregnant. Quarantine of any introduced animal was done in 28.4% of the farms and most of them kept such animals apart from the herd for less than 30 days. The availability of paddocks destined to keep sick animals away from healthy animals was found on 40.7% of the farms.

Related to reproductive practices, 54.5% answered that they adopted natural mating practices and 80.2% reported the use of artificial insemination (AI) and bulls simultaneously. When IA was used, 57% of the farms reported that it was always the same person that provided the service, 18% said to have more than one person inseminating the animals, and 42.4% of the farms, the service was provided by a veterinary company (Figure below). Farms that routinely used rectal examination for pregnancy diagnostic represent 41.8% of the study population, and the ones that noticed that the responsible for doing the procedure did not changed rubber gloves between/among different animals were 40.6% of the total farms. In 61.2% of the farms, the origin of the bulls was related to an external source, i.e., they were not born in the property. Among the farmers that keep a bull as permanent member of the herd, 37% had bought the animal and 24.2% just borrowed the animal from a nearest neighbor.


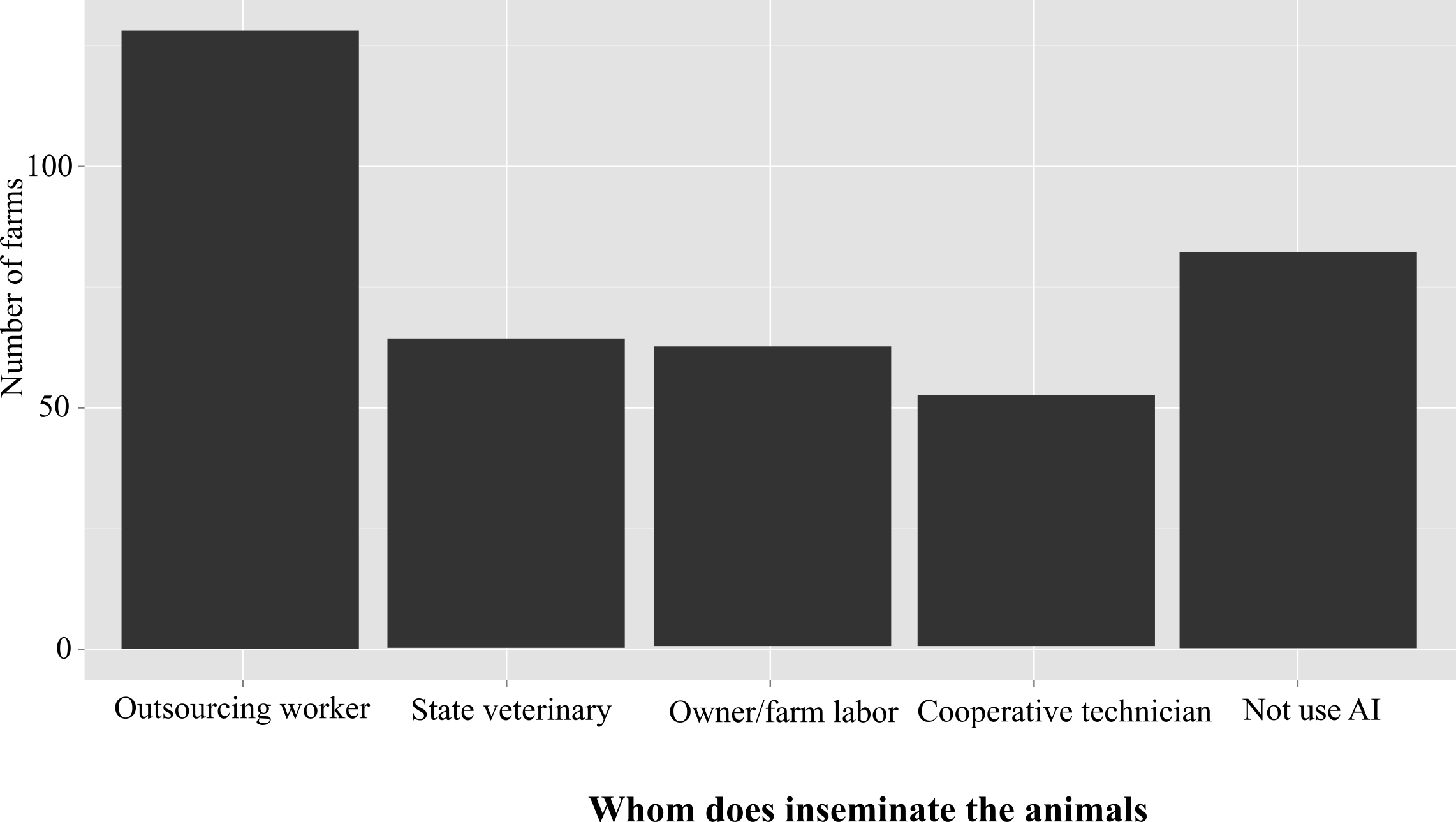


Artificial insemination been performed by different knowledge persons.

About calving, in 90.7% of the answers it occurred out on the fields. Answers related to the management after calving showed that 43% of the farms did not allowed the calves to suck colostrum and were immediately separated from their mothers, while 39% of the farms were keeping colostrum stock (a pool of milk from many cows to feed on all new calves). The rise of weak newborns and abortion occurrence was observed in 31.4% e 25.5% of the farms, respectively.

The use of the same needle in diffent animals were praticated on 78.5% of the farms, which may be involved on the spread of many diseases including BVDV. The possibility of a direct contact over the fences lines between/among animals from neighboring farms was reported as positive in 55.4% of the answers. On the herd management, 59.4% of owners divided animals by age in different fields for feeding purpose. The collapse of fence and movement of animals’ between/among farms was reported to happen sometimes on 42.3% of the farms.
