# Supplementary material for: What variables are important in predicting bovine viral diarrhea virus? A random forest approach
Source: Vet Res. 2015 Jul 24;46(1):85. doi: 10.1186/s13567-015-0219-7 (PMC4513962; doi:10.1186/s13567-015-0219-7)
Supplement: Additional file 2: Frequency of important predictor variables. — The prevalence of important predictor variables obtained by serological assay results provides details of disease occurrence in the study population. [file 13567_2015_219_MOESM2_ESM.doc]

**Additional file 2. The frequency or central tendency measurement of important predictor variables**

| Variables | Frequency (%)or median | |
| --- | --- | --- |
|  | BVDV (**+)** | BVDV (-) |
| (1) Who inseminates the animals  Owner/farm labor  State veterinary  Cooperative technician  Outsourcing worker | 30 (45.59)  8 (11.76)  10 (14.71)  19 (27.94) | 32 (13.58)  56 (23.05)  42 (17.28)  112 (46.09) |
| (2) Number of neighboring farms that have cattle | 1 | 1 |
| (3) What proportion of the farm income is based on milk production |  |  |
| 0-20%  21-40%  41-60%  61-80%  81-100% | 11 (11.96)  17 (18.48)  20 (21.74)  23 (25.00)  21 (22.83) | 64 (21.84)  45 (15.36)  78 (26.62)  38 (12.97)  68 (23.21) |
| (4) For how many years has the farm produced milk | 17 | 19 |
| (5) Frequency of technical assistance |  |  |
| Annual | 1 (2.38) | 5 (3.09) |
| Semester | 6 (14.29) | 19 (11.73) |
| Monthly | 17 (40.48) | 54 (33.33) |
| Only when needed | 18 (42.86) | 84 (51.85) |
| (6) Is rectal palpation performed routinely  No  Yes | 34 (30.63)  59 (53.15) | 192 (65.08)  103 (34.92) |
| (7) Number of different inseminators in the last year | 1 | 1 |
| (8) What is the origin of the bulls |  |  |
| Born in the farm | 41 (50.00) | 64 (40.25) |
| Purchased | 18 (21.95) | 55 (34.59) |
| Borrowed | 23 (28.05) | 40 (25.16) |
| (9) Frequency of veterinary assistance |  |  |
| Annual | 3 (3.53) | 1 (0.38) |
| Semester | 2 (2.35) | 2 (0.75 |
| Monthly  Only when requested | 25 (29.41)  55 (64.71) | 38 (14.29)  225 (84.59) |
| (10) Are animals placed in a quarantine before introduction |  |  |
| No | 25 (25.77) | 80 (76.19) |
| Yes | 17 (17.53) | 25 (23.81) |
| (11) What is the origin of animals brought into the farm |  |  |
| Own produced animals | 39 (41.94) | 169 (57.29) |
| Only purchased or exchanged | 3 (3.23) | 11 (3.73) |
| Mix (own **and** purchased or exchanged) | 51 (54.84) | 115 (38.98) |
| (12) How often does the fence between/among farms that hold cattle collapse |  |  |
| Never | 40 (43.01) | 156 (52.88) |
| Sometimes | 44 (47.31) | 120 (40.68) |
| Always | 9 (9.68) | 19 (6.44) |
| (13) How the cows are milked |  |  |
| Manual | 9 (9.68) | 44 (14.92) |
| Semi-automatic | 41 (44.09) | 169 (57.29) |
| Automatic | 43 (46.24) | 82 (27.80) |
| (14) Was there an increase in abortions |  |  |
| No | 65 (69.89) | 224 (75.93) |
| Yes | 28 (30.11) | 71 (24.07) |
| (15) Does calving occur in closed barns |  |  |
| No | 76 (81.72) | 220 (74.58) |
| Yes | 17 (18.28) | 75 (25.42) |
| (16) Number of cows lactating at the sampling moment | 17 | 10 |
| (17) Were animals vaccinated for BVDV |  |  |
| No | 85 (91.40) | 449 (84.98) |
| Yes | 8 (8.90) | 44 ( 15.02) |
| (18) Was there a rise of mating failure |  |  |
| No | 63 (74) | 203 (68.81) |
| Yes | 30 (32.26) | 92 (31.19) |
| (19) Do animals share the same feed and water containers |  |  |
| No | 17 (25.37) | 46 (22.66) |
| Yes | 50 (74.63) | 157 (77.34) |
| (20) Number of cows not lactating at the sampling moment | 17 | 10 |
| (21) Is colostrum stock available |  |  |
| No | 55 (59.14) | 182 (61.69) |
| Yes | 38 (40.86) | 113 (38.31) |
| (22) Total farm area in hectares  < 0.2 Km^2^  > 0.21 Km^2^ | 40 (43.01)  53 (56.99) | 162 (54.92)  133 (45.08) |
| (23) Are paddocks available for sick animals |  |  |
| No | 56 (60.22) | 174 (58.98) |
| Yes | 37 (39.78) | 121 (41.02) |
| (24) Who administers the medications |  |  |
| Employer of the med store | 6 (8.82) | 5(2.06) |
| Veterinary | 25 (36.76) | 28 (11.52) |
| Technician | 8 (11.76) | 56 (23.05) |
| Neighboring/friend | 10 (14.71) | 42 (17.28 |
| Farm Owner | 19 (27.94) | 112 (46.09) |
| (25) Is blood from a sick animal injected into the healthy ones |  |  |
| No  Yes | 70 (76.92)  21 (23.08) | 279 (96.54)  10 (3.46) |
