# Supplementary figures and images for: What variables are important in predicting bovine viral diarrhea virus? A random forest approach
Source: Vet Res. 2015 Jul 24;46(1):85. doi: 10.1186/s13567-015-0219-7 (PMC4513962; doi:10.1186/s13567-015-0219-7)

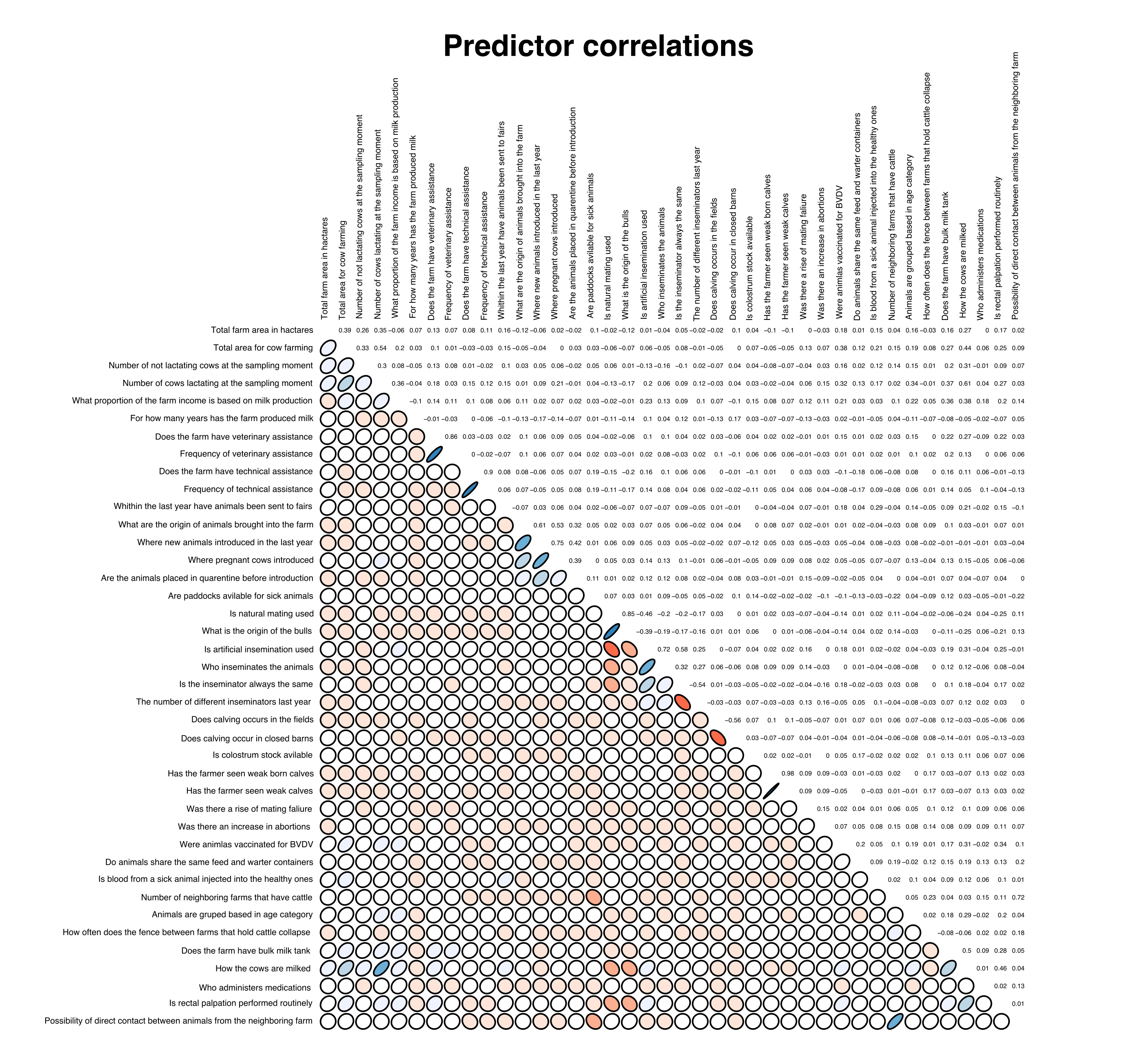

Supplement: Additional file 3: Correlation matrix for predictor variables. — Negative correlation is represented by red ellipses pending to the left; positive correlation is represented by blue ellipses pending to the right. The exact correlation values are given in the upper panel. [file 13567_2015_219_MOESM3_ESM.docx]
