# Supplementary material for: What variables are important in predicting bovine viral diarrhea virus? A random forest approach
Source: Vet Res. 2015 Jul 24;46(1):85. doi: 10.1186/s13567-015-0219-7 (PMC4513962; doi:10.1186/s13567-015-0219-7)
Supplement: Additional file 4: Models performance for 10 randomly generated independent test data sets. — The AUC scores are computed for 10 repetitions of model training and testing. In each repetition, a random portion of 80% of data is used for training, and the remaining 20% for testing (independent data). [file 13567_2015_219_MOESM4_ESM.docx]

**Additional file 4 Models performance for 10 randomly generated independent test data sets.** The AUC scores are computed for 10 repetitions of model training and testing. In each repetition, a random portion of 80% of data is used for training, and the remaining 20% for testing (independent/external data). The best performance in each repetition is shown in boldface. The mean and standard deviations for each classifier after the 10 repetitions are also reported. We observe that RF achieves the best performance in six repetitions, while GBM provides the best model in the remaining four. The AUC scores for the worst and best model trained with RF are 0.6925 and 0.8764, while GBM’s worst and best performance are 0.6178 and 0.8180. This corresponds to a performance gain of 12.09% and 7.13% in the worst and best cases, respectively.

|  | **RF** | **SVM** | **GBM** |
| --- | --- | --- | --- |
| Repetition 1 | **0.6925** | 0.6388 | 0.6178 |
| Repetition 2 | **0.8170** | 0.7480 | 0.7873 |
| Repetition 3 | **0.6944** | 0.6800 | 0.6388 |
| Repetition 4 | **0.7198** | 0.6522 | 0.6762 |
| Repetition 5 | 0.7447 | 0.6704 | **0.7557** |
| Repetition 6 | **0.7303** | 0.6954 | 0.7279 |
| Repetition 7 | **0.8764** | 0.7576 | 0.8180 |
| Repetition 8 | 0.7112 | 0.7155 | **0.7595** |
| Repetition 9 | 0.7749 | 0.6867 | **0.7902** |
| Repetition 10 | 0.7049 | 0.6379 | **0.7298** |
| **Mean** | 0.7466 | 0.6883 | 0.7301 |
| **SD** | 0.0598 | 0.0419 | 0.0666 |
